# Wafer-scale Thermodynamically Stable GaN Nanorods via Two-Step Self-Limiting Epitaxy for Optoelectronic Applications

**DOI:** 10.1038/srep40893

**Published:** 2017-01-18

**Authors:** Hyun Kum, Han-Kyu Seong, Wantae Lim, Daemyung Chun, Young-il Kim, Youngsoo Park, Geonwook Yoo

**Affiliations:** 1 Semiconductor R&D Center Samsung Electronics Hwasung, South Korea; 2 School of Electronic Engineering Soongsil University Seoul, South Korea

## Abstract

We present a method of epitaxially growing thermodynamically stable gallium nitride (GaN) nanorods via metal-organic chemical vapor deposition (MOCVD) by invoking a two-step self-limited growth (TSSLG) mechanism. This allows for growth of nanorods with excellent geometrical uniformity with no visible extended defects over a 100 mm sapphire (Al_2_O_3_) wafer. An *ex-situ* study of the growth morphology as a function of growth time for the two self-limiting steps elucidate the growth dynamics, which show that formation of an Ehrlich-Schwoebel barrier and preferential growth in the c-plane direction governs the growth process. This process allows monolithic formation of dimensionally uniform nanowires on templates with varying filling matrix patterns for a variety of novel electronic and optoelectronic applications. A color tunable phosphor-free white light LED with a coaxial architecture is fabricated as a demonstration of the applicability of these nanorods grown by TSSLG.

Wide bandgap semiconductor gallium nitride (GaN) is a promising III-V material for a wide range of advanced optical and electrical device applications such as LEDs, solar cells, lasers, single photon sources, and power devices^1^,^2^,^3^,^4^,^5^,^6^. It is also possible to monolithically integrate GaN devices with silicon technology to further enhance the functionality of conventional CMOS devices^7^. Both catalyst-assisted and catalyst-free growth of III-V nanorods have been demonstrated and studied in various epitaxial growth systems in terms of growth morphology and uniformity^6^,^8^,^9^,^10^. Growth of nanostructures practically eliminates extended defect formations (e.g. dislocations, stacking faults, and twins) which arise due to growth on foreign substrates with widely different lattice constants. These nanostructures also allow utilization of various crystalline planes and confinements in a single device and supports devices with three-dimensional architectures^11^,^12^. For III-V materials such as GaN, growth on foreign substrates (typically sapphire or silicon carbide for LED applications) is a practical necessity due to the high cost of bulk GaN substrates. Many groups have studied nanorod growths using molecular beam epitaxy (MBE) and/or metal-organic chemical vapor deposition (MOCVD) with pulsed-growth method to form the nanorods^13^,^14^,^15^,^16^,^17^,^18^,^19^,^20^,^21^. Growth using these tools and methods, however, take a relatively long time to grow, and the use of a catalyst such as Au particles induces growth of nanowires in random locations with non-uniform shape, height, and length dimensions. This may potentially preclude opportunities for monolithic integration with other devices as well as cause general issues with consistency and reliability. It is, therefore, desirable to develop a method of quickly forming uniform nanorods with precise position control of the nanorod growth. In this regard, selective area epitaxy (SAE) of nanostructures is attractive since it allows for precise control of the position of the nanorods, enhancing consistency and manufacturability. To grow identical nanorods using conventional SAE, however, requires that the geometrical parameters of the pattern matrix, such as the shape, size, and pitch, be the same since the source flow and temperature gradient must be the same within the substrate surface. Another issue is that for growth of GaN on large lattice mismatched substrates such as sapphire or silicon, the typical threading dislocation (TD) density is high (~10^8^ cm^−2^). The growth rate near the vicinity of the TDs is much faster than on the planar surface, leading to areas of nanorods with stark contrast in terms of geometrical size with the rest of the growth region. Overcoming this problem would further expand the applicability of the selective area growth technique, as well as provide a more controllable GaN nanorod template for novel applications. To nullify these issues, we developed a two-step self-limited (TSSL) growth method of creating thermodynamically stable wurtzite GaN nanorods with minimal geometrical variations or extended defects such as threading dislocations or stacking faults. This method provides the means of growing extremely uniform nanorods over a 100 mm sapphire substrate (scalable to larger substrates) with a large growth condition window and excellent repeatability. With this technique, it is possible to form uniform nanorods even with variations in the size and pitch of the underlying growth mask. The growth formation mechanism of the nanorods is meticulously observed *ex-situ* to provide a clear understanding of the growth dynamics.

## Experimental

**Figure 1:**
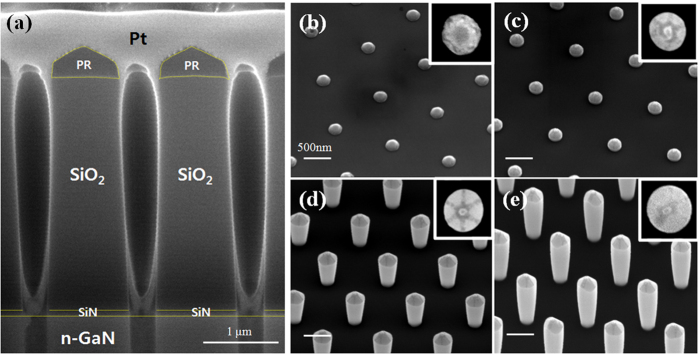
(**a**) Cross-section SEM image of the filling matrix prepared by focused-ion beam milling (FIB). A thin Pt layer is deposited on top to maintain the photoresist (PR) profile during the FIB process. A 30° tilt view of GaN growth inside the filling matrix is shown in 5 minute increments in (**b**)–(**e**). The insets show a top-view image of the nanorod.

Wafer preparation before the TSSL growth begins with deposition of 100 nm SiN layer followed by a 2.9 μm of SiO_2_ on a sapphire substrate using PECVD at 300 °C. Approximately 2 μm of Si-doped n-type GaN is grown on sapphire before depositing the dielectric layers. For this particular study, we used an ASML PAS 300 KrF lithography tool to create circular holes with a diameter of 300 nm in hexagonal patterns (SI, Figure S1). Three regions, denoted R, G, and B, were defined with different pitch between the holes of 2 μm, 1.4 μm, and 1 μm, respectively. The pattern was then etched using deep reactive-ion etching techniques using C_4_F_8_, Ar, and O_2_ gases. A detailed report on the etch chemistry and resulting etch profiles can be found in our previous publication^22^. The etch chemistry is carefully chosen to allow the Si-doped GaN layer to work as an effective etch stop. A slight over etch is performed to prepare a clean GaN surface free of SiN particles at the bottom of the holes for epitaxial growth. To check we did not have any re-deposition of SiN on the SiO_2_ sidewalls, we employed an optical emission spectroscopy end-point detection (EPD) system which allows *in-situ* monitoring of materials being etched in the chamber. It is true that some re-deposition may have occurred at the sidewalls which EPD may not detect. However, the amount of re-deposition is trivial, as evidenced by the uniform shape of the sidewalls of our nanorods during the GaN filling step. Moreover, GaN growth on SiN surface is energetically unfavorable, thus would not affect the growth in a meaningful manner. At this point, the preparation for growth is complete, and the SiN/SiO_2_ stack constitutes the filling matrix. Figure 1(a) shows a profile SEM image of the completed growth mask. Due to the high aspect ratio of the diameter to the thickness of the dielectric mask, the holes are etched in a semi-anisotropic manner, where the diameter at the bottom is approximately 100 nm smaller than the diameter at the top. This tapered etch profile, as seen later, will not affect the formation of perfectly hexagonal GaN nanorods. A schematic of the fabrication flow is giving in the SI (Figure S2).

The template is then transferred to an MOCVD reactor (Veeco Gen 3), and the first step of the TSSL growth is carried out. The substrate temperature is ramped up to 1120 °C under an ammonia (NH_3_) overpressure of 2 L at a chamber pressure of 100 mbar. Once the temperature is stabilized, tri-methyl gallium (TMGa) is injected for GaN growth. A flow of 20 sccm TMGa is flown until the patterned hole is nearly filled in the R region, where the time it takes to nearly fill the hole was calculated from several calibration growths in advance. It is also possible to dope the nanorod at this stage by flowing the relevant dopant precursors such as silane (SiH_4_) or bis(cyclopentadienyl)magnesium (Cp_2_Mg) for n- and p-type doping, respectively. To match the doping of our substrate GaN layer, we introduced SiH_4_ as our n-type doping source. The gas flow is carefully controlled by a high-precision Horiba digital mass-flow controller (MFC), which can control gas flow within ±1% of the set point. The temperature is controlled and monitored by a thermocouple within the reactor, as well as an emissivity corrected pyrometry for *in-situ* substrate temperature monitoring of the substrate surface developed by LayTec. It should be noted that since the G and B regions have smaller pitch, the volume to fill in with the precursors are greater. Thus, the B region will be slightly less filled than the G region, and both B and G regions will be filled less than the R region.

## Results and Discussion

**Figure 2:**
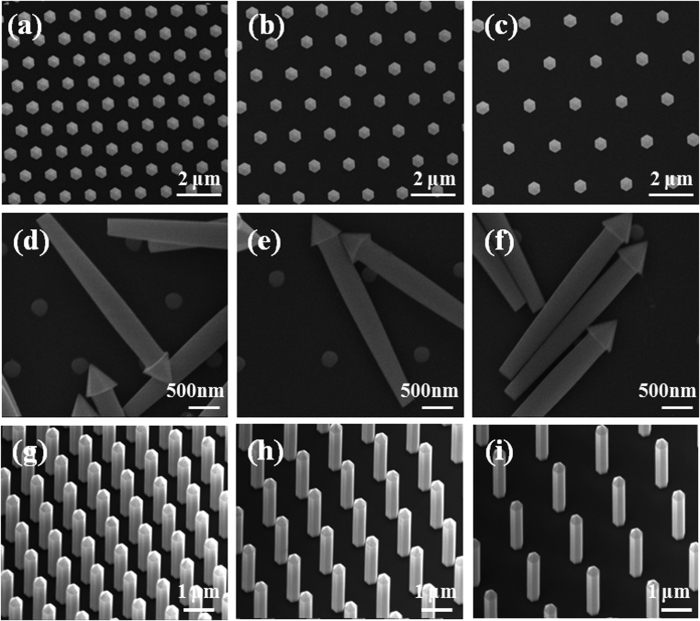
Top view SEM image after the first self-limiting growth step for (**a**) B, (**b**) G, and (**c**) R regions. The nanorod profile after removing the SiO_2_ filling matrix for (**d**) B, (**e**) G, and (**f**) R regions. A 30° tilt view after the second self-limiting growth step for (**g**) B, (**h**) G, and (**i**) R regions.

The growth morphology as a function of time of the nanorod tip inside the mask is shown in Fig. 1(b). Each picture was taken after 5 minutes of growth using SEM. A similar study was recently done by Gačević *et al*.^23^ where the initial nucleation and tip formation of GaN pyramids were observed and modeled. Initially, nucleation starts on the surface of the GaN buffer layer, which then quickly fills the mask while conforming to the shape of the hole. No growth is observed on the SiO_2_ surface since the preferential nucleation domain is on the crystalline GaN surface rather than on SiO_2_. The tip of the rod inside the hole initially nucleates in what resembles a hexagon on a spherical cap, but quickly loses its morphology as due to the tendency to minimize its free surface energy which is spatially constrained by the sidewalls of the mask hole. At this stage, the growth rate (or the filing rate of the etched hole) is linear with respect to the V/III ratio. Once the filling is complete, GaN starts to grow outside the filling matrix, forming a perfectly hexagonal pyramid structure. This is clearly shown in Fig. 2(a)–(c) for B, G, and R regions, which consists of six m-plane edges and r-plane surfaces. This is due to the thermodynamically favorable m-facet growth of GaN, and is the most thermodynamically stable form in our growth temperature range^24^.

After the first part of the two-step growth is compete, the wafer is taken out of the MOCVD chamber and dipped into a dilute HF (10:1) solution for 20 minutes to remove the entire SiO_2_ layer. The SiN layer has a relatively low etch rate of (20 nm/min) compared to SiO_2_ and, therefore, is used as an etch stop layer. It also acts as an electrical isolation for each individual nanorod. The nanorods were broken for a profile SEM view, as shown in Fig. 2(d)–(f) for all three regions. The sidewall surface of the nanorods have no crystallographic preference and follow the profile of the SiO_2_ mask, and thus energetically unstable at this point. The surface of the nanorod tip, however, is in a very stable r-plane configuration. The stability can be tested by increasing the growth time and observing the change in geometrical features such as the diameter, height, and crystallographic orientation of the tip. We observed no measureable change in any of the geometrical or crystallographic features with an increase of growth time from 15 minutes to 45 minutes.

**Figure 3:**
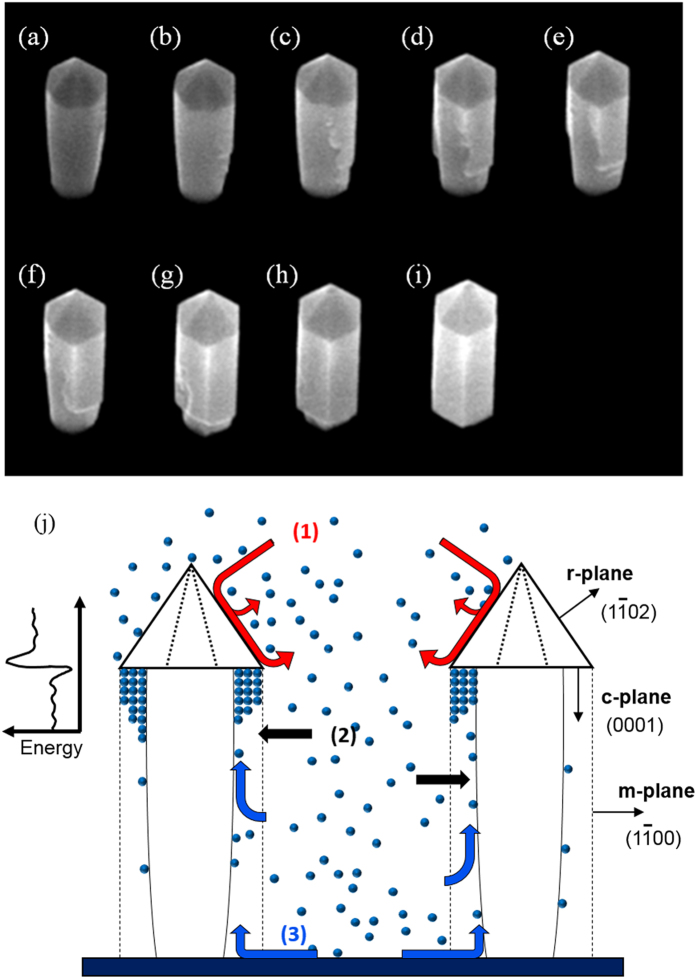
(**a**)**–**(**i**) A tilt-view (30°) SEM image of a single nanorod during the second self-limiting regrowth step at an interval of approximately 3 minutes. We note that it is an image of the same nanorod grown next to a pre-defined alignment mark on the wafer. (**j**) A schematic illustration of the adatom dynamics during the second self-limiting growth step, which consists primarily of (1) impingement from above, (2) impingement from the side, and (3) surface diffusion from the substrate floor. The inset on the top left shows a schematic illustration of a Ehrlich-Schwoebel barrier formed at the neck of the nanorod.

With the first step ensuring a thermodynamically stable tip, it is possible to proceed to the second step of forming energetically stable sidewalls. The sample is once again loaded into an MOCVD chamber for regrowth. The temperature was ramped up to 1150 °C with 2 L of NH_3_ overpressure at a chamber pressure of 100 mbar. Once at a growth temperature of 1150 °C, TMGa flow of 20 sccm was flown for a total of 15 minutes. The rods after regrowth are shown in Fig. 2(g)–(i), which reveals a fully crystalline GaN nanorod with m-plane sidewalls and an r-plane hexagonal tip for all three R, G, and B regions. Once again, increasing the growth time from 15 minutes to 45 minutes did not alter the dimensions or crystallographic properties of the nanorods (SI, Figure S3). Also, no growth on the SiN surface was observed. Figure 3(a) shows the growth morphology as a function of time for the nanorod regrowth step for a total of 30 minutes in approximately 3 minute intervals. For this study, the growth rate was reduced by lowering the TMGa flow from 20 to 5 sccm to get a better image of the morphological change of the nanorod between each time step. We note that the SEM images are from the same nanorod formed next to a premade alignment mark, and are a representation of all nanowires grown on the wafer. We observed that the growth proceeds beneath the hexagonal tip, and moves downward along the axial direction of the rod. It is evident that the driving growth dynamics is governed by the thermodynamic stability of the hexagonal pyramid tip, *i.e.* the dimensions of the nanorod are fixed by the dimensions of the pyramidal tip. The source dynamics involved in the regrowth step consists of (1) source impingement and diffusion from above the nanorod, (2) direct impingement of sources to the sidewall, and (3) surface diffusion of the adatoms along the SiN surface to the sidewall, as illustrated in Fig. 3(b). Since the tip is thermodynamically stable, the impinging source is desorbed and flown to adjacent nanorods. The thermodynamically unstable sidewalls become an energetic sink for Ga adatoms. More specifically, due to the presence of a step, there exists an Ehrlich-Schwoebel barrier in-between the base of the pyramidal tip and the “neck” of the nanorod. The barrier energy is illustrated in the inset of Fig. 3(j). Due to the presence of this barrier, we can deduce that the majority of the adatoms for growth are from direct impingement of sources to the sidewall and surface diffusion of adatoms from the SiN surface up the rod sidewall. Moreover, it is known that growth of c-planes are energetically favorable in comparison to the m- or r-plane in wurtzite GaN (i.e. the growth rate, G/R, of the c-plane is much faster than m- or r-plane, G/R_c_ >> G/R_m_, G/R_r_). This results in a layer by layer growth in the -c-plane direction down the nanorod sidewall, as evidenced in Fig. 3.

**Figure 4:**
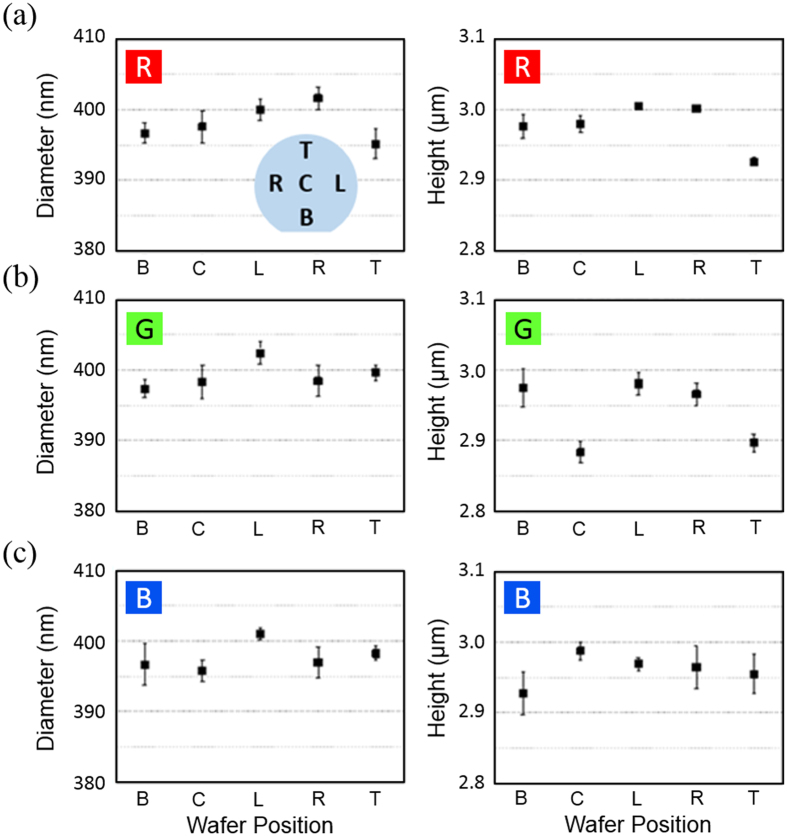
(**a**)–(**c**)The diameter and height distribution of the grown nanorods over a 100 mm wafer for each R, G, and B region, respectively. The inset shows the positions measured on the wafer.

Once the entire structure becomes thermodynamically stable, growth stops and most adatoms are desorbed from the wafer. This leaves very uniform rods that could be used as a platform for a variety of applications. Figure 4 shows the nanorod diameter and height uniformity within a single 100 mm wafer for each R, G, and B region. The diameter of the rod, which is defined as the length between two opposite vertices, falls within ±5 nm. The height distribution follows the distribution of the SiO_2_ thickness, which ranges between 2.9~3.0 μm from the base of the nanorod to the apex of the hexagonal pyramid. This variation in nanorod uniformity over a 100 mm wafer is, to our knowledge, unprecedented. More than twenty full wafers were grown and measured in various positions of the wafer (center, left, right, top, and bottom) to verify wafer-to-wafer and in-wafer uniformity of the grown nanorods.

**Figure 5:**
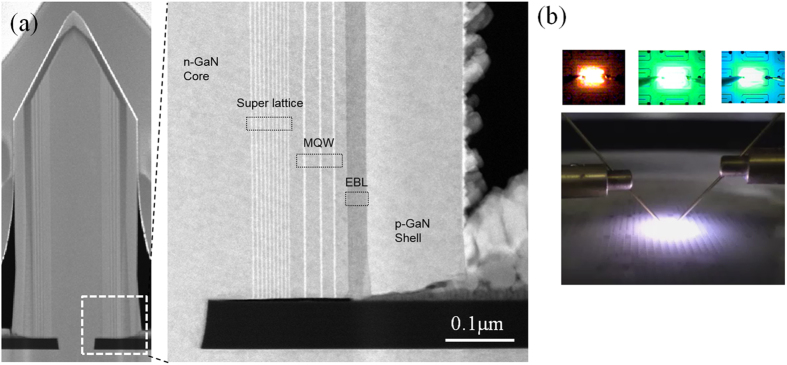
(**a**) A TEM image of a single core-shell nanorod with the full LED layers grown, consisting of 10-pairs of InGaN/GaN super-lattice, 3-pairs of InGaN/GaN MQW active region, and a single AlGaAs EBL, followed by a Mg-doped p-GaN shell. (**b**) A microscopy image of fabricated nanorod LEDs emitting at various wavelengths individually (top), and monolithically (bottom).

As a demonstration of the quality and applicability of the structures grown, monolithic phosphor-free white emitting InGaN/GaN core-shell light-emitting diodes were fabricated. Several advantages of nanorod-based LED is the reduced quantum confined stark effect (QCSE), which reduces the electron-hole wave function overlap, ultimately leading to reduced radiative efficiency and power droop as a function of injection current. The QCSE effect is caused by an internal piezoelectric field resulted from the biaxial strain between InGaN and GaN lattice difference^25^. By utilizing the non-polar m-plane of GaN, it is possible to eliminate the QCSE effect, leading to reduced efficiency droop and increased radiative efficiency. Another advantage is the increase in active area for the same chip footprint. Also, by carefully engineering the pitch of the nanorod cores, it is possible to monolithically integrate red, green, and blue emission regions on a single chip, allowing white light without the need of phosphor by virtue of indium species conservation. This further enhances the LED characteristics by removing the phosphor conversion efficiency, as well as improving device reliability which may be caused by phosphor degradation due to prolonged exposure to internally generated heat. Such nanorod and phosphorless LED devices are actively being developed at both academic and industry labs due to its possibility of out-performing its planar counterpart^8^,^26^,^27^,^28^. The shell layer consists of 10-pairs of InGaN/GaN super-lattice for strain relief, 3-paris of InGaN/GaN multi-quantum well and barrier layers, AlGaN electron blocking layer to prevent electron overflow at high current densities, and a p-GaN layer for hole carrier injection. The growth conditions for the shell layers and fabrication steps are reported elsewhere^22^. A TEM image is shown in Fig. 5(a) for a single nanorod. No extended defects such as threading dislocations were observed in the TEM image. Over 20 rods were analyzed in this manner, and further study using HR-TEM at the regrowth interface is being done. Moreover, the formation of dimensionally uniform cores across all red/green/blue regions is highly desired for proper operation of nanorod LEDs; dimensional uniformity is a contributing factor that affects the carrier flow dynamics in the device, which ultimately determines the emission properties as well as key electrical performance such as operating voltage and droop. Devices were fabricated (630 × 970 μm^2^) using conventional photolithography, metal deposition, and etch techniques. As shown in Fig. 5(b), each of the R, G, and B regions show red, green, and blue emission at 65 mA current bias (1.5 A/cm^2^), with a wavelength of 600 nm, 510 nm, and 467 nm, respectively. The color rendering index and color temperature can be controlled by changing the ratio of each RGB area and the pitch between the rods in each region. A more detailed study on the LED chip characteristics based on TSSL nanorods will be reported in a separate manuscript.

## Conclusion

In conclusion, we have presented a method of fabricating GaN nanorods with wafer-level uniformity using a two-step self-limited method via MOCVD. Thermodynamically stable nanorods with diameter and height variations within ±5 nm and ±0.5 μm, respectively, are grown over a 100 mm sapphire wafer. The growth morphology show that energetically stable GaN planes reduce the growth rate of the rods significantly once the planes are formed, allowing for the growth of identical nanorods dimension-wise, which can then be used as a template for a variety of wide-bandgap device applications. A color tunable phosphor-free white emitting LED is fabricated as a demonstration of the applicability of the nanorods grown via TSSLG.

## Additional Information

**How to cite this article**: Kum, H. *et al*. Wafer-scale Thermodynamically Stable GaN Nanorods via Two-Step Self-Limiting Epitaxy for Optoelectronic Applications. *Sci. Rep.* **7**, 40893; doi: 10.1038/srep40893 (2017).

**Publisher's note:** Springer Nature remains neutral with regard to jurisdictional claims in published maps and institutional affiliations.

## Supplementary Material

Supplementary Information
