# Supplementary material for: Wafer-scale Thermodynamically Stable GaN Nanorods via Two-Step Self-Limiting Epitaxy for Optoelectronic Applications
Source: Sci Rep. 2017 Jan 18;7:40893. doi: 10.1038/srep40893 (PMC5241666; doi:10.1038/srep40893)
Supplement: Supplementary Information [file srep40893-s1.doc]

**Supplementary Information**

Wafer-scale Thermodynamically Stable GaN Nanorods via Two-Step Self-Limiting Epitaxy for Optoelectronic Applications

Hyun Kum^1^, Han-Kyu Seong^1^, Wantae Lim^1^, Daemyung Chun^1^, Yong-il Kim^1^, YoungSoo Park^1^, Geonwook Yoo^2^

^1^Semiconductor R&D Center

Samsung Electronics

Hwasung, South Korea

^2^School of Electronic Engineering

Soongsil University

Seoul, South Korea


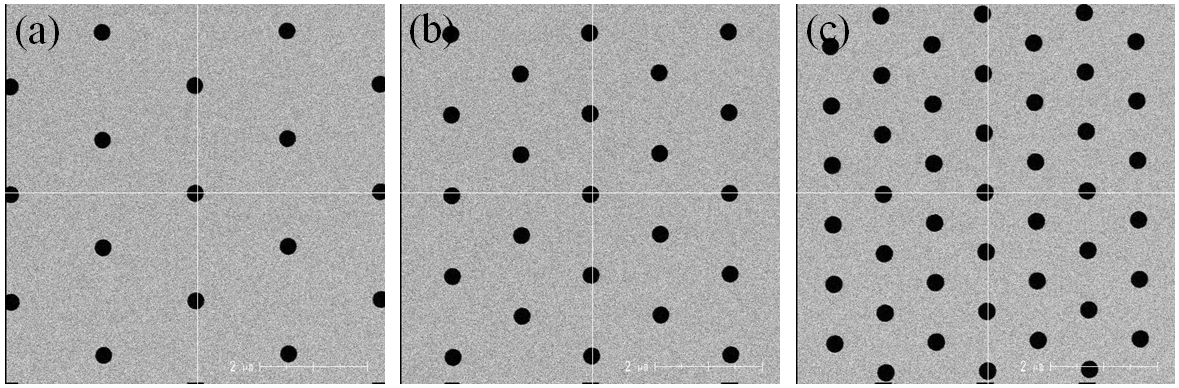


**Figure S1.** A Hitachi CD-SEM image of the SiN/SiO_2_ mask patterned with ASML KrF lithography tool for (a) R, (b) G, and (c) B regions.


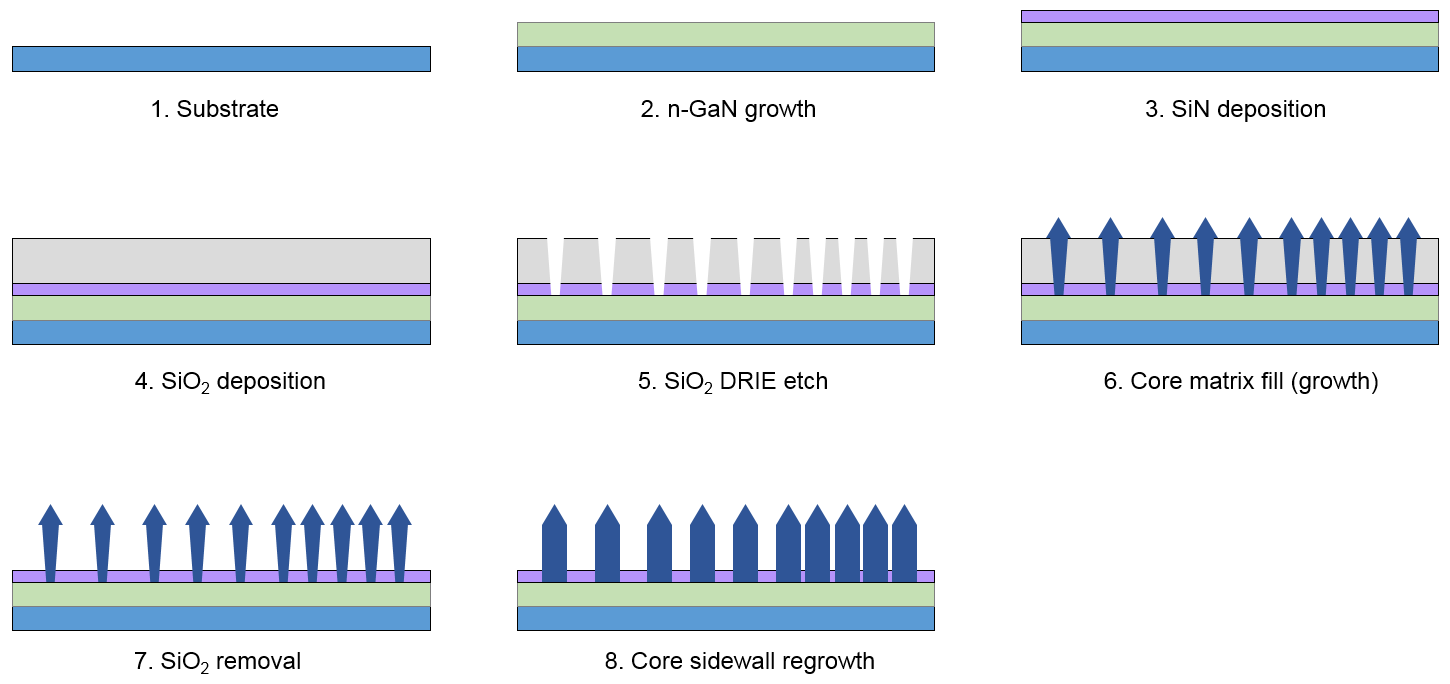


**Figure S2.** Full growth and fabrication steps for the two-step self-limited thermodynamically stable GaN nanorods.


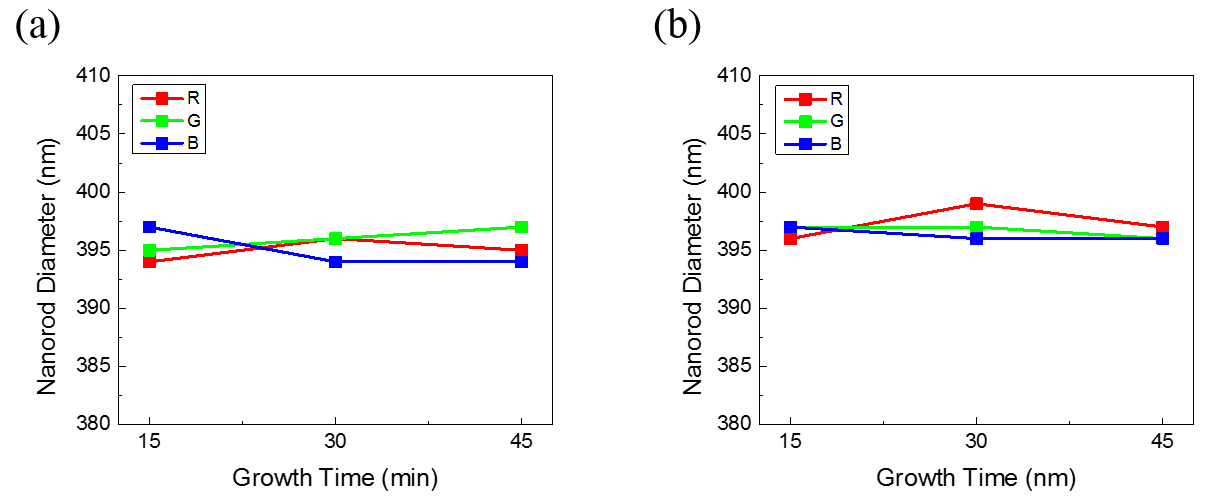


**Figure S3.** Change in nanorod diameter as a function of growth time during (a) core matrix fill (step 6 in Figure S2), and (b) core sidewall regrowth step (step 8 in Figure S2) for each R, G, and B regions of the filling matrix.
